# Hyper-Cross-Linked Polystyrene as a Stabilizing Medium for Small Metal Clusters

**DOI:** 10.3390/molecules26175294

**Published:** 2021-08-31

**Authors:** Alexey V. Bykov, Galina N. Demidenko, Linda Zh. Nikoshvili, Lioubov Kiwi-Minsker

**Affiliations:** 1 Department of Biotechnology, Chemistry and Standardization, Tver State Technical University, A. Nikitina str. 22, 170026 Tver, Russia; bykovav@yandex.ru (A.V.B.); xt345@mail.ru (G.N.D.); 2 Regional Technological Centre, Tver State University, Zhelyabova str. 33, 170100 Tver, Russia; 3 Department of Basic Sciences, Ecole Polytechnique Fédérale de Lausanne, ISIC-FBS-EPFL, CH-1015 Lausanne, Switzerland

**Keywords:** palladium, platinum, clusters, hyper-cross-linked polystyrene, benzene adsorption

## Abstract

Among different polymers nanostructured cross-linked aromatics have the greatest potential as catalytic supports due to their exceptional thermal and chemical stability and preservation of the active phase morphology. This work studies the ability of hyper-cross-linked polystyrene (HPS) to stabilize small Pd_n_ and Pt_n_ (*n* = 4 or 9) clusters. Unrestricted DFT calculations were carried out for benzene (BZ) adsorption at the BP level of theory using triple-zeta basis sets. The adsorption of BZ rings (stepwise from one to four) was found to result in noticeable gain in energy and stabilization of resulting adsorption complexes. Moreover, the interaction of metal clusters with HPS micropores was also addressed. For the first time, the incorporation of small clusters in the HPS structure was shown to influences its geometry resulting in the stabilization of polymer due to its partial relaxation.

## 1. Introduction

The development of new hybrid catalytic systems is of high importance, since it can lead to significant increases in the efficiency of catalytic reactions. Active centers formed in a polymeric environment combine the advantages of both homogeneous (selectivity, activity) and heterogeneous (easy separation) catalysts. Depending on the types of catalytically active metal and of polymeric support, the catalytic properties of such systems can be readily tuned.

Highly porous hyper-cross-linked aromatic polymers (HAP) presenting high mechanical, thermal and chemical stability are commercially available [1]. Such amorphous microporous polymers are promising for catalysis [2], e.g., as supports for both Pd complexes and Pd^0^ nanoparticles (NPs) [3,4,5,6,7] due to their high specific surface area, developed porosity and dual hydrophilic-hydrophobic character [7,8,9,10,11,12]. At the same time, functional groups on the polymer enhance the metal dispersion, and the specific porous structure ensures fast diffusion of reactants to the active centers. The distinctive feature of HAP is the simplicity of their production and relatively low price. Also due to the variety of available monomers, the pore sizes can be controlled and varied [13,14,15]. It is important to underline that the control of HAP porosity is crucial for high efficiency of the resulting catalyst and is achieved by sufficient chemical cross-linking, which prevents the collapse of polymeric chains into a dense non-porous material [16,17].

During the last decades the interest of both the industrial and scientific communities in hyper-cross-linked polystyrene (HPS) has greatly increased. This is because of its advantages over traditional macroporous resins and activated carbons in terms of higher sorption capacity and easy regeneration [18]. Due to its high degree of cross-linking, which can exceed 100%, the HPS consists of rigid nanovoids (“nanopores”) serving as nanoreactors for metal particle growth [19,20,21].

Recently we have found that small Pd_n_ (*n* ≤ 13) clusters can be also formed by the XPS method on the HPS surface along with Pd^0^ NPs [22]. It is well known that HPS is an effective stabilizing medium for Pd^0^ NPs, even used for complex reactions such as Suzuki cross-coupling, which involve homogeneous stages in the catalytic cycle [23]. However, the role in cross-coupling reaction of small Pd_n_ clusters formed during the preliminarily catalyst activation with gaseous hydrogen is still unclear: either they possess catalytic activity, or they just serve as a depot for active metal; either they influence the polymer properties (e.g., thermal and mechanical stability), or remain inert in the HPS micropores. To answer these questions, first the strength of interaction needs to be found, i.e., the energy of the adsorption of the small metal clusters (Pd and Pt) on the aromatic rings of HPS. It is important to determine also the mutual influence of metal clusters and polymeric chains.

The dynamic nature of catalytically active species has become evident in heterogeneous catalysis [24,25,26,27]. However, there are no direct instrumental methods to assess small metal clusters immobilized on supports, and hence, mechanistic studies are necessary. DFT modeling is widely used to describe polymers and metal clusters [28,29,30,31,32]. Many works describe DFT modeling of Pd_n_ clusters [33,34,35,36] as well as adsorption of different compounds on them [37,38,39]. Duca et al. [40] have calculated the geometry of HPS and Pd_n_ (*n* ≤ 9) clusters using the CAM-B3LYP functional joined with the LANL2-DZ basis set and found that hosting HPS deforms when Pd atoms are added to growing cluster. The balance between the Pd-C and the Pd-Pd interactions was shown to be crucial, and Pd_4_ is on the borderline. At the same time it was proposed that the benzene (BZ) rings of HPS allow stabilization of Pd_n_ clusters, and small cavities in HPS structure affect both the geometric and the electronic properties of Pd clusters [40]. Nevertheless, there is still lack of data on the interaction of HPS and small metal clusters, i.e., the energy of adsorption of polymer BZ rings on Pd and Pt clusters.

Carneiro et al. [41] have studied BZ adsorption on Pd and Pt clusters using B3LYP/LANL2DZ. For the most stable triplet state of the Pd_7_ cluster the energy of BZ adsorption was found to be about 26–29 kcal/mol. It is noteworthy that B3LYP is the most popular density functional [36,37,38,39,40,41], which fits well for geometry optimization. At the same time, it was mentioned [42] that B3LYP is not fully suitable for energetic calculations. Hence some authors [42,43] preferred PBE functional. In order to resolve the issue of the functional choice, we performed calculation of BZ adsorption on Pd_4_ cluster in a triplet state while using several available exchange-correlation functionals (Figure S1). We have found that BP functional provides values of the adsorption energy close to the results of temperature-programmed desorption of BZ adsorbed on Pd(111) [44,45], and also fits well some other reported results [43,46,47].

This work studies the interaction of immobilized small Pd and Pt clusters with the HPS micropores, and the degree of micropore deformation during the cluster adsorption. Thus, for the first time, in the framework of this work we carried out DFT modeling of BZ adsorption (both free BZ rings and BZ of HPS micropore) on small clusters of Pd_n_ (*n* = 4 or 9) and Pt_4_ using BP functional. As a result the adsorption energies of BZ rings on Pd and Pt clusters were calculated, helping in the analysis of real catalytic experiments over HAP impregnated by noble metals. It is noteworthy that some works has studied small Pd clusters confined in the HPS [40], but the stabilization of the microporous polymeric network by metal clusters has been never revealed. For the first time the difference between Pt_4_ and Pd_4_ stabilized into the HPS micropores was shown.

## 2. Results

Unrestricted DFT calculations were carried out at the BP level of theory for small metal clusters (Pd_4_, Pd_9_, Pt_4_, and Pt_9_) and the adsorption of one, two, three and four BZ rings on them, as well as for the adsorption of these clusters in the model HPS micropore to study the stabilization of palladium and platinum clusters in aromatic polymeric networks.

### 2.1. Adsorption of Aromatics on Pd_n_ Clusters

**Figure 1:**
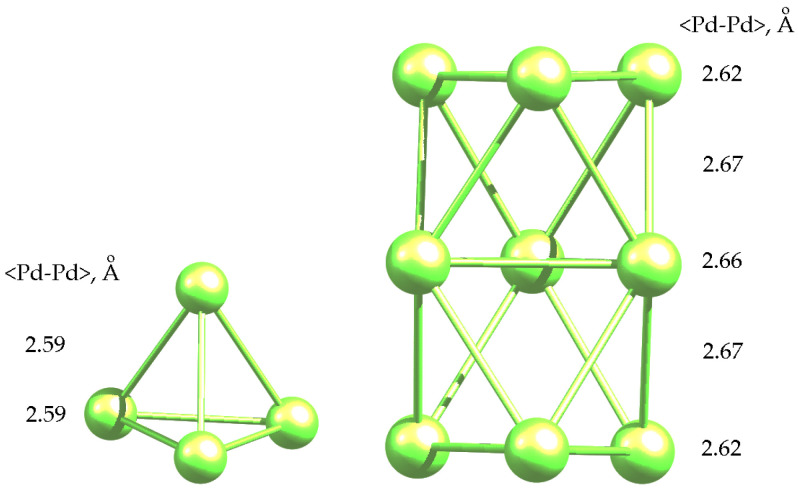
Optimized geometries of Pd_4_ and Pd_9_ clusters in a triplet state.

The results of the calculations of the ground state of palladium clusters are presented in Table 1 and Figure 1.

As it can be seen, triplet was the ground state for both clusters, which is in good agreement with the literature data [40,41,42,43]. The average <Pd-Pd> bond lengths were 2.59 Å and 2.65 Å for Pd_4_ and Pd_9_ clusters, respectively. These values were used to estimate the changes in the bond lengths of clusters in adsorption complexes. It is noteworthy that in the structure of Pd_4_ cluster, which was a slightly distorted tetrahedron, two mutually non-intersecting edges had a length of 2.55 Å, while all the other edges had a length of 2.61 Å. Obviously, such a distortion was due to the Jahn-Teller effect. In the case of BZ adsorption on Pd_4_ clusters, the triplet was also shown to be the ground state (Table 2).

**Figure 2:**
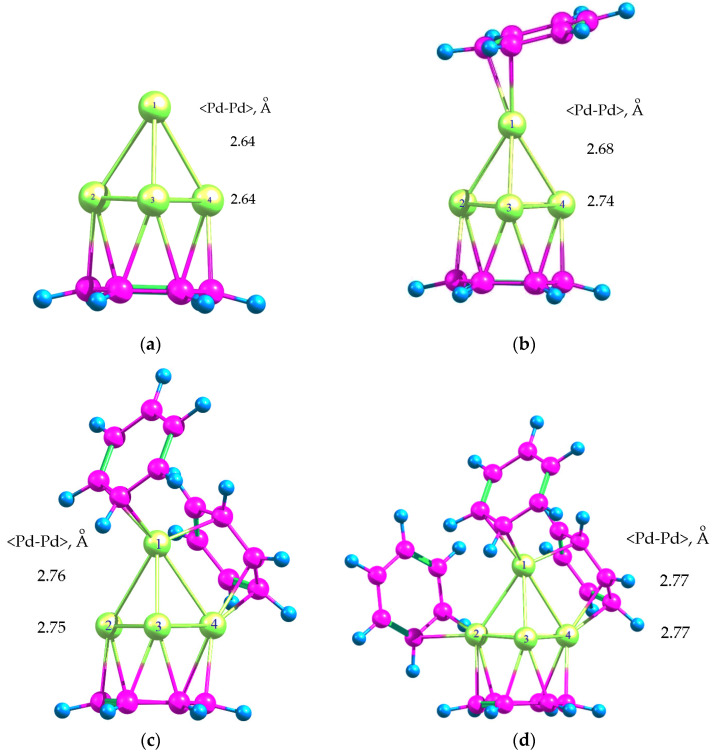
Optimized geometries of Pd-BZ adsorption complexes: Pd_4_*C_6_H_6_ (**a**), Pd_4_*2C_6_H_6_ (**b**), Pd_4_*3C_6_H_6_ (**c**) and Pd_4_*4C_6_H_6_ (**d**).

Calculation of Pd_4_ adsorption complexes in the triplet state with one, two, three and four BZ molecules (Figure 2) has shown that Pd_4_ is able to coordinate all the four BZ rings: the adsorption energies in the series Pd_4_*C_6_H_6_, Pd_4_*2C_6_H_6_, Pd_4_*3C_6_H_6_ and Pd_4_*4C_6_H_6_ were −145.9, −200.4, −256.6 and −286.1 kJ/mol, respectively. The adsorption of Pd_4_ with its base, consisting of three Pd atoms, on the first BZ ring led to the gain in energy of −149.5 kJ/mol (or −48.6 kJ/mol per each of the three Pd atoms). At the same time, the average <Pd-Pd> bond length increased by 1.9% (Figure 2a).

The adsorption of the second and third BZ rings on Pd_4_ led to an energy gain of −54.5 kJ/mol and −56.2 kJ/mol, respectively, due to BZ coordination with the single vertex Pd atom (Figure 2b) and also along the edge of the cluster with two Pd atoms already involved in coordination with other BZ rings (Figure 2c). In the latter case, this corresponded to the change in energy of the system by −28.1 kJ/mol per each of two Pd atoms. Moreover, the coordination of the second and third BZ rings also resulted in the increase of the average <Pd-Pd> bond length up to 2.71 Å (Figure 2b) and 2.75 Å (Figure 2c) for Pd_4_*2C_6_H_6_ and Pd_4_*3C_6_H_6_, respectively. This corresponds to an increase of the average <Pd-Pd> bond length by 4.6% and 6.2% as compared to free Pd_4_ cluster. It is noteworthy that the average <Pd-Pd> distance between the atoms of base was greater than those for free Pd_4_ cluster by 5.6% (in Pd_4_*2C_6_H_6_) and 5.9% (in Pd_4_*3C_6_H_6_).

The adsorption of fourth BZ molecule led to an even greater expansion of the cluster: the average <Pd-Pd> bond length increased up to 2.77 Å, which is by 7.0% higher than for the free cluster. In this case, the disproportion between the average <Pd-Pd> distance of the vertex atom and <Pd-Pd> distance of the base practically disappeared.

Coordination of palladium atoms belonging to Pd_4_ cluster with BZ rings caused partial transfer of electron density from palladium to carbon (Table 3) and its redistribution to π-electron density of aromatic ring (see also Figure S2).

**Figure 3:**
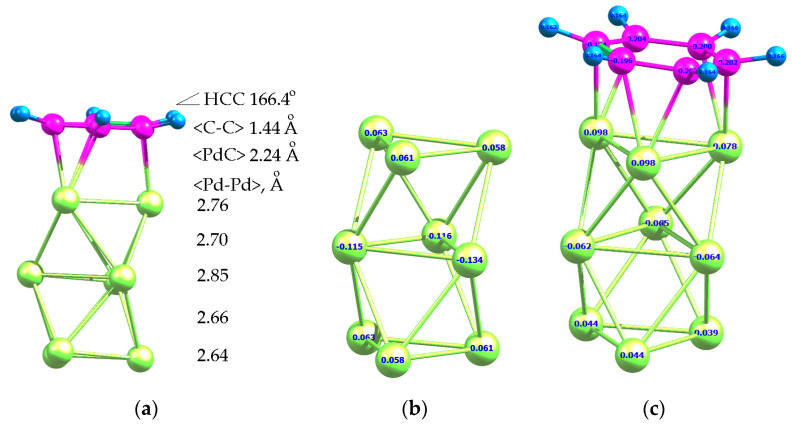
Optimized geometry of Pd_9_*C_6_H_6_ adsorption complex (**a**) and Löwdin charges of Pd_4_ (**b**) and Pd_9_*C_6_H_6_ (**c**).

As a next step, the geometry of Pd_9_*C_6_H_6_ adsorption complex (Figure 3a) and the adsorption energy of BZ molecule on free Pd_9_ cluster were calculated. The adsorption energy of BZ on Pd_9_ was found to be −97.4 kJ/mol, i.e., the energy of BZ adsorption decreased with the increase of cluster size. The electron density was also transferred from Pd_9_ to the carbons of BZ (Figure 3b,c).

**Figure 4:**
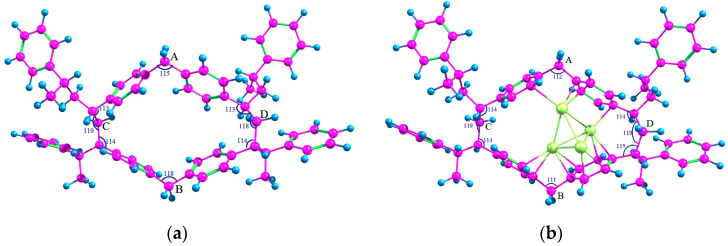
Optimized geometry of HPS micropore before (**a**) and after (**b**) incorporation of Pd_4_.

Based on the results obtained, it is possible to assume that Pd_4_ clusters while being in their ground state are able to coordinate BZ molecules and to be stabilized in the aromatic environment. This fact can serve as a reference point for evaluating and comparing the efficiency of the interaction between palladium clusters and the polymer micropores. It is known that noble metal clusters confined in polymers are too large to be investigated by using first principle calculations [32]. Hence it is common practice to replace whole polymers with similar smaller molecules. To study the possibility of stabilization and to find the adsorption energy of Pd_4_ cluster in the HPS network, a model micropore, which is formed by the fragments of polystyrene chains whose BZ rings are cross-linked with methylene bridges, as well as the adsorption complex of Pd_4_ cluster in this pore, were calculated at the same level of theory. In each case, the entire structure was allowed to relax during the optimization. The calculated structures are shown in Figure 4.

The adsorption energy was found to be −237.3 kJ/mol (or −59.3 kJ/mol per each palladium atom), which is quite close to the adsorption energy of three BZ molecules on a Pd_4_ cluster. The average <Pd-Pd> bond length was increased by 6.2% as compared to a free Pd_4_ cluster that coincides with the change of bond length for Pd_4_*3C_6_H_6_ system.

The slight decrease of the adsorption energy of Pd_4_ in the polymer micropores as compared to the case of BZ adsorption on Pd_4_ was likely due to the limitations of the conformational mobility of the polymer linkages, which did not allow optimizing the geometry of the adsorption complex the same way as in the case of individual BZ rings.

To determine the changes in geometry of the polymer micropores during the incorporation of Pd_4_ cluster, the methylene groups, indicated in Figure 4 as “A”, “B”, “C” and “D”, were selected. Obviously, the most significant changes in the pore structure occurred in the area of the cluster adsorption (Table 4) since Pd_4_ itself is much smaller than the pore and cannot fill completely the entire micropore. Incorporation of Pd_4_ resulted in the increase in the pore diameter (“AB”) by 4.6%, which is associated with the adaptation of the pore geometry during the coordination of BZ rings with the cluster. At the same time, the valence angles of covalent bonds of the methylene groups “A” and “ B” decreased from 115° and 118° to 112° and 111°, respectively, which indicates the decrease in pore tension (the angles tend to 109°28′, corresponding to sp^3^-hybridized carbon atoms). Significant changes in other distances (“BD” and “AD” by 3.4 and 2.1%) are associated with conformational changes that do not affect the valence angles of methylene groups.

**Figure 5:**
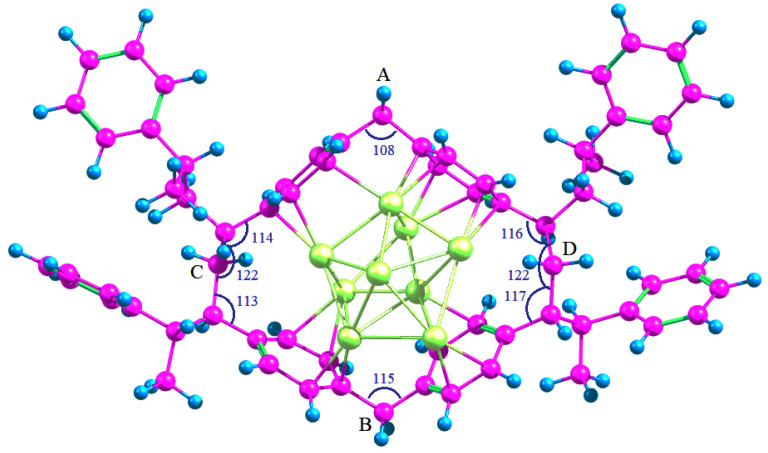
Optimized geometry of Pd_9_ incorporated into the HPS micropore.

Incorporation of Pd_9_ (see Figure 1) into the same micropore caused dramatic restructuring of the cluster (Figure 5). The adsorption energy of Pd_9_ was −341.2 kJ/mol, which was 44% higher than in the case of Pd_4_ incorporated into the same micropore. However, an average value of the adsorption energy calculated per each Pd atom was −37.9 kJ/mol, which was 36% lower as compared to Pd_4_ stabilized in the same micropore (−59.3 kJ/mol per atom).

**Figure 6:**
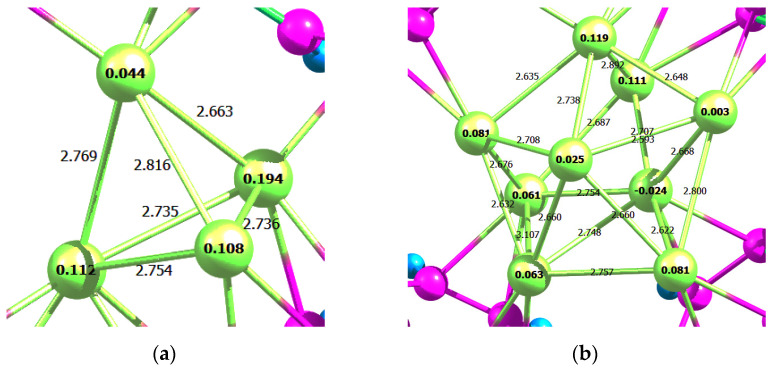
Löwdin charges and bond lengths for optimized polymer-stabilized Pd_4_ (**a**) and Pd_9_ (**b**).

In the case of both Pd_4_ and Pd_9_, there was a shift in the electron density from palladium to BZ rings of the polymer (Figure 6).

It is noteworthy that the change in the pore geometry is directly associated with the removal of tension in the methylene bridges “A” and “B”: elongation of the pore along the “AB” direction by 14.8% (Table 5) and decrease of valence angles of the methylene groups “A” and “B (Figure 5) can be seen.

### 2.2. Adsorption of Aromatics on Pt_n_ Clusters

**Figure 7:**
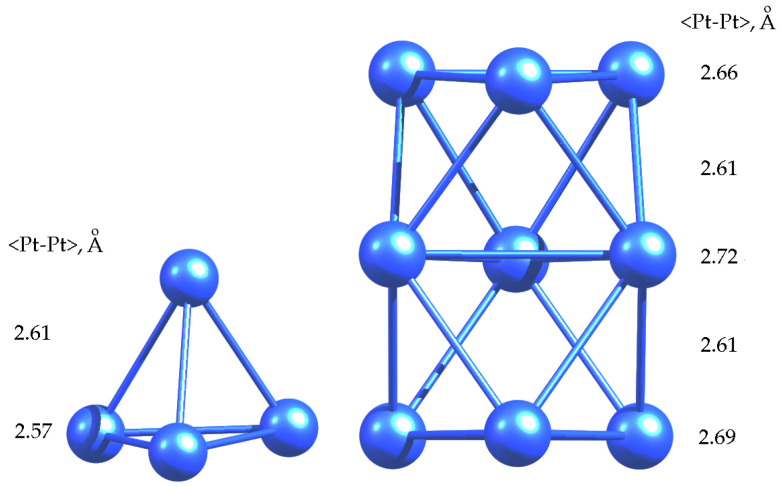
Optimized geometries of Pt_4_ and Pt_9_ clusters in a triplet state.

The results of calculation of the ground state of platinum clusters are presented in Table 6 and Figure 7. Similarly to the Pd_n_ clusters, the triplet one was found to be the ground state for Pt_4_. The average <Pt-Pt> bond length is 2.59 Å and 2.65 Å for Pt_4_ and Pt_9_ cluster, respectively. Moreover, the structure of Pt_4_ cluster was slightly distorted: one edge of tetrahedron had length of 2.69 Å, while all the other edges are 2.57–2.58 Å.

Similarly to Pd_4_*C_6_H_6_ (see Table 2), the triplet was the ground state in the case of Pt_4_*C_6_H_6_ (Table 7).

**Figure 8:**
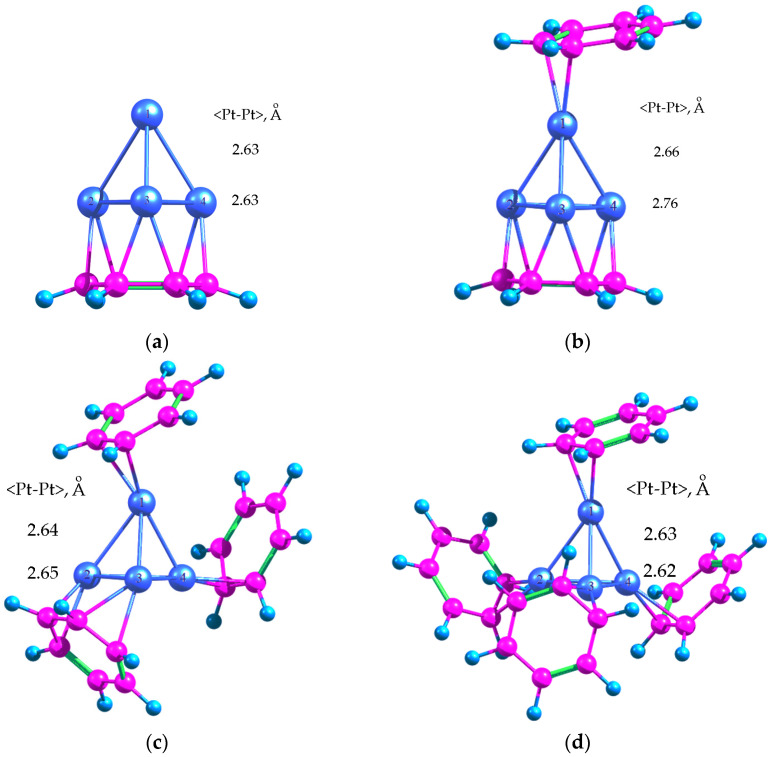
Optimized geometries of Pt-BZ adsorption complexes: Pt_4_*C_6_H_6_ (**a**), Pt_4_*2C_6_H_6_ (**b**), Pt_4_*3C_6_H_6_ (**c**) and Pt_4_*4C_6_H_6_ (**d**).

Calculation of Pt_4_ adsorption complexes in the triplet state with one, two, three and four BZ molecules (Figure 8) revealed that the adsorption energies in the series Pt_4_*C_6_H_6_, Pt_4_*2C_6_H_6_, Pt_4_*3C_6_H_6_ and Pt_4_*4C_6_H_6_ were −230.6, −273.8, −387.6, and −463.0 kJ/mol, respectively. Thus the adsorption of Pt_4_ with its base on the first BZ ring led to the gain in energy of −230.6 kJ/mol (or −76.9 kJ/mol per each Pt atom). The average <Pt-Pt> bond length increased by 1.5% (Figure 8a).

The adsorption of the second and third BZ rings on Pt_4_ led to energy gains of −43.2 kJ/mol and −113.8 kJ/mol, respectively. The second BZ ring was coordinated with the single vertex Pt atom (Figure 8b) similarly to Pt_4_*2C_6_H_6_ (Figure 2b), while the addition of the third BZ ring resulted in noticeable rearrangement of the coordination ensemble (Figure 8c). At the same time the average <Pt-Pt> bond length increased up to 2.71 Å (Pt_4_*2C_6_H_6_) and to 2.65 Å (Pt_4_*3C_6_H_6_), which corresponded to an increase of the average <Pt-Pt> bond length by 4.6% and 2.1% as compared to free Pt_4_ cluster. The average <Pt-Pt> distance between the atoms of base was greater than those for free Pt_4_ cluster by 6.6% (in Pt_4_*2C_6_H_6_) and 2.3% (in Pt_4_*3C_6_H_6_). Such slight changes in <Pt-Pt> bond length in the case of Pt_4_*3C_6_H_6_ are likely due to the observed rearrangement of the ensemble.

The adsorption of fourth BZ ring led to additional gain in energy by −75.4 kJ/mol and to slight compression of the cluster: the average <Pt-Pt> bond length in the adsorption complex Pt_4_*4C_6_H_6_ was 2.63 Å, which is only by 1.4% higher than for free Pt_4_ cluster and can be explained by the relatively symmetrical environment of the cluster by BZ rings.

In contrast to palladium, when the platinum atoms coordinated with BZ molecules, partial transfer of the electron density from carbon atoms to Pt occurred (Table 8) due to redistribution of π-electron density of BZ rings (see Figure S3). The higher the number of adsorbed BZ rings, the more electron density is transferred to a Pt_4_ cluster.

**Figure 9:**
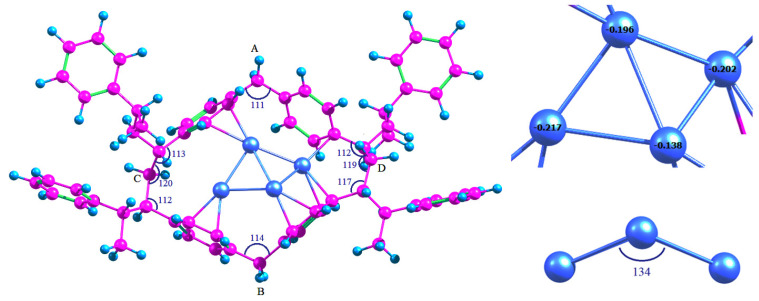
Optimized geometry of Pt_4_ incorporated into the HPS micropore and Löwdin charges of Pt atoms.

The possibility of stabilization of Pt_4_ cluster in the HPS micropore was also studied using the same model micropore as in the case of Pd_n_ clusters (see Section 2.1) at the same level of theory. The calculated structure is shown in Figure 9. The adsorption energy was −371.3 kJ/mol, which was slightly lower than in the case of Pt_4_*3C_6_H_6_ adsorption complex. Due to the fact that the adsorption energy of BZ rings on Pt_4_ cluster was relatively high, and the polymer pore had rather rigid structure with limited conformational mobility, complete restructuring of the cluster and its adaptation to the pore geometry was observed. The average value of <Pt-Pt> bond length was 2.60 Å at the significant transfer of electron density from the polymer to Pt (see the Löwdin charges in Figure 9).

The pore itself was noticeably expanded in the direction of methylene groups “A” and “B” (Table 9): the distance “AB” increased by about 11%. This also caused the decrease of tension of the methylene groups “A” and “B” since their valence angles decreased from 115° and 118° to 111° and 114°, respectively. At the same time, some tension appeared in the methine radical located on the segment “BD” between the BZ ring and the methylene group “D”.

## 3. Discussion

DFT calculations of BZ adsorption (from one up to four rings) on the small Pd_n_ and Pt_n_ (*n* = 4) clusters were carried out at the BP level of theory, which allowed obtaining refined values of the adsorption energies serving as starting point for calculation of metal clusters interaction with the micropore of the aromatic polymer (HPS). It was shown that Pd_4_ and Pt_4_ clusters can be effectively stabilized due to the adsorption of BZ rings. Such adsorption leads to geometric distortions of the metal clusters as well as to charge redistribution between the clusters to their chemical environment. It was found for the first time that charge transfer in the case of palladium and platinum has contrary direction: in the case of Pd_4_ the partial transfer of electron density from the metal to carbon takes place, while in the case of Pt_4_, in contrary, the partial transfer of electron density occurs from carbon atoms of BZ rings to the metal. It is noteworthy that interaction of Pt_4_ cluster with BZ rings was stronger and caused higher gain in energy (the energy of adsorption complex Pt_4_*3C_6_H_6_ was −273.8 kJ/mol) as compared to Pd_4_ (the energy of adsorption complex Pd_4_*3C_6_H_6_ was −256.6 kJ/mol).

Clusters of both types (Pd_4_ and Pt_4_) were found to be effectively stabilized in a model micropore of the polymer (HPS) calculated at the same level of theory. It was shown that the metal clusters can be captured by the HPS micropores and retained with a significant (essential for the purposes of heterogeneous catalysis) adsorption energies. The adsorption energy of Pd_4_ in the HPS micropore was −237.3 kJ/mol, and the increase of Pd_n_ cluster size up to *n* = 9 (Pd_9_) resulted in the energy gain (−341.2 kJ/mol), which confirms that the growth of small metal clusters in the aromatic polymeric environment is the energy beneficial process accompanied by the adaptation of clusters’ geometry to the micropore structure. At the same time, in the case of platinum the interaction of Pt atoms with the BZ rings of the HPS micropore is so strong that it causes noticeable distortion of clusters’ geometry even for Pt_4_.

Moreover, it was shown for the first time that the incorporation of small metal clusters into the HPS micropores changes the pores’ geometry, which in turn decreases the methylene bridge (the cross-links of HPS) tension. Thus one can conclude that the noble metal clusters act as micropore stabilizers, which may prevent the polymer destruction during significant vibrations and pulsations of its chains. The latter can take place during high-temperature treatment of polymer-based composites. Such a treatment is commonly applied in heterogeneous catalysis as a part of the activation procedure, i.e., gas-phase reduction of catalysts in a hydrogen flow. Though it is not possible to directly assess small metal clusters buried in the pores of HAP, the results obtained in this study can be useful to explain behavior of real catalytic systems exposed to high-temperatures.

Finally, it can be concluded that the high adsorption ability of Pd_4_ and Pt_4_ clusters in the HPS polymeric network can lead to their deposition in micropores or small mesopores, which can have a strong impact on catalytic properties of such systems. While being stabilized in aromatic environment, small metal clusters can be inaccessible for reagents during heterogeneous catalytic reactions or alternatively they can play a role of buffer for catalytically active metal species. The interaction of metal clusters incorporated into micropores of HAPs with different catalytic substrates should be further studied and this work is in progress.

## 4. Computational Methods

The search for the level of theory for calculations was performed on the basis of calculations of the energy of BZ adsorption on Pd_4_ cluster in the triplet state. The adsorption energy was calculated by the unrestricted methods of Hartree-Fock, perturbation theory, as well as the DFT using B3LYP, PBE and BP functionals both with and without the ZORA formalism. The effect of the empirical van der Waals correction on the adsorption energy in the case of DFT was also tested. The results of calculations are presented in Figure S1. Since up to date there are no experimental data on the structure of palladium clusters and the adsorption energies of arenes on palladium clusters, the value of the adsorption energy of BZ on Pd (111) surface found from calorimetric studies [44] is one of few reliable sources of information for validation. This value (−131 kJ/mol) was taken as a reference point. Based on the comparison of the obtained results and the impossibility of verifying the validity of the empirical van der Waals correction for the considered systems, all the presented studies were performed under conditions that better describe the experimental result.

All the DFT calculations were unrestricted and were performed using Orca 4.2.1 [48,49] package at the BP level of theory. The relativistic effects were taken into account by means of the ZORA formalism. For C and H the ZORA-def2-TZVP basis set was used [50], including with diffusion and polarized sets. In case of palladium, old-ZORA-TZVP basis set was used. In case of platinum, segmented all-electron relativistically contracted, SARC-ZORA-TZVP basis set was used. During each optimization, the entire structure was allowed to relax. Adsorption energies were calculated as follows:

***E_ads_*** = ***E_ABn_*** − ***E_A_*** − ***nE_B_*** (1)

where ***E_ABn_*** is the energy of adsorption complex; ***E_A_*** is the metal cluster energy; ***E_B_*** is the energy of BZ.

## 5. Conclusions

In the framework of this study, BZ adsorption on small clusters of Pd_n_ and Pt_n_ (*n* = 4 or 9) was calculated at the BP level of theory. The adsorption of arenes (and BZ, in particularly) was previously studied in many works, but we obtained the adsorption energies for the metal cluster interactions with the polymer (HPS) chains at the same level of theory. For the purpose of catalysis the calculation of adsorption energies of noble metal clusters is of great importance as it can serve as a starting point for further calculations of reactants’ adsorption and the migration of noble metals within polymeric networks. We have confirmed that the B3LYP functional, which was used in most previous works, not always provides accurate calculations in comparison with BP and PBE functionals. Thus, using the BP functional we compared for the first time the adsorption of BZ molecules on clusters of palladium and platinum, which allowed us to conclude that these metals behave differently in an aromatic environment, i.e., opposite charge transfer directions were found. Moreover, Pt_4_ clusters were found to undergo dramatic changes in geometry when encapsulated in the micropores of HPS. The stabilization of the polymer micropores by Pt and Pd clusters was also revealed for the first time, which represents a very important result for the preparation of heterogeneous polymer-based catalysts.

## Figures and Tables

**Table 1:** Results of geometry and relative energy calculations for Pd_4_ and Pd_9_.

Pd_4_		Pd_9_			
2S+1	dE (eV)	<Pd-Pd> (Å)	2S+1	dE (eV)	<Pd-Pd> (Å)
1	0.452	2.59	1	0.215	2.65
3	0.000	2.59	3	0.000	2.65
5	1.082	2.58	5	0.049	2.57

**Table 2:** Relative energies of Pd_4_*C_6_H_6_ and the energy of BZ adsorption on Pd_4_ for different states.

2S+1	dE, eV	E_ads_, kJ/mol ^(1)^
1	0.263	−164.1
3	0.000	−145.9
5	2.126	−45.2

^(1)^ calculated with respect to Pd_4_ of the same multiplicity.

**Table 3:** Löwdin charges of Pd_4_ and Pd-BZ adsorption complexes.

Location (Sequential Number of Pd Atom)	Pd_4_	Pd_4_*C_6_H_6_	Pd_4_*2C_6_H_6_	Pd_4_*3C_6_H_6_	Pd_4_*4C_6_H_6_
vertex (1)	0.000	0.056	0.042	0.044	0.057
base (2)	0.000	0.058	0.103	0.135	0.142
base (3)	0.000	0.058	0.100	0.113	0.124
base (4)	0.000	0.057	0.098	0.154	0.161

**Table 4:** Structure parameters of the polymer micropore in case of Pd_4_ incorporation.

Distance, Å	Polymer	Pd_4_*Polymer	Change, %
AB	7.83	8.19	4.6
CD	10.72	10.90	1.7
AC	6.67	6.71	0.6
AD	6.71	6.85	2.1
BC	6.76	6.78	0.3
BD	6.75	6.98	3.4

**Table 5:** Structure parameters of the polymer micropore in case of Pd_9_ incorporation.

Distance, Å	Polymer	Pd_9_*Polymer	Change, %
AB	7.83	8.99	14.81
CD	10.72	10.32	−3.73
AC	6.67	6.79	1.80
AD	6.71	6.91	2.98
BC	6.76	6.74	−0.30
BD	6.75	6.93	2.67

**Table 6:** Results of geometry calculations for Pt_4_ and Pt_9_.

Pt_4_		Pt_9_			
2S+1	dE, eV	<Pt-Pt>, Å	2S+1	dE, eV	<Pt-Pt>, Å
1	0.496	2.61	1	-	-
3	0.000	2.59	3	0.000	2.65
5	0.038	2.58	5	-	-

**Table 7:** Relative energies of Pt_4_*C_6_H_6_ and the energy of BZ adsorption on Pt_4_ for different states.

2S+1	dE, eV	E_ads_, kJ/mol ^1^
1	0.392	−240.6
3	0.000	−230.6
5	1.145	−123.8

^1^ calculated with respect to Pt_4_ of the same multiplicity

**Table 8:** Löwdin charges of Pt_4_ and Pt-BZ adsorption complexes.

Location (Sequential Number of Pt Atom)	Pt_4_	Pt_4_*C_6_H_6_	Pt_4_*2C_6_H_6_	Pt_4_*3C_6_H_6_	Pt_4_*4C_6_H_6_
vertex (1)	0.000	−0.079	−0.206	−0.174	−0.167
base (2)	0.000	−0.278	−0.106	−0.112	−0.160
base (3)	0.000	−0.279	−0.107	−0.111	−0.162
base (4)	0.000	−0.279	−0.102	−0.178	−0.164

**Table 9:** Structure parameters of the polymer micropore in case of Pt_4_ incorporation.

Distance, Å	Polymer	Pt_4_*Polymer	Change, %
AB	7.83	8.69	10.98
CD	10.72	10.39	−3.08
AC	6.67	6.69	0.30
AD	6.71	6.78	1.04
BC	6.76	6.66	−1.48
BD	6.75	7.07	4.74

## Data Availability

Data sharing is not applicable to this article.

## Supplementary Materials

The following are available online, Figure S1: Comparison of different calculation methods and basis sets for Pd_4_*C_6_H_6_ adsorption complex, Figure S2: Isosurfaces 0.06 e/Å^3^ of the adsorption complexes Pd_4_*C_6_H_6_, Pd_4_*2C_6_H_6_, Pd_4_*3C_6_H_6_ and Pd_4_*4C_6_H_6_, Figure S3: Isosurfaces 0.08 e/Å^3^ of the adsorption complexes Pt_4_*C_6_H_6_, Pt_4_*2C_6_H_6_, Pt_4_*3C_6_H_6_ and Pt_4_*4C_6_H_6_, Table S1: Coordinates of atoms of the optimized structures in the ground state.

## Sample Availability

Not available.

## Abbreviations

BZ: benzene; NP, nanoparticle; HAP, hyper-cross-linked aromatic polymer; HPS, hyper-cross-linked polystyrene.
