# Supplementary material for: Hyper-Cross-Linked Polystyrene as a Stabilizing Medium for Small Metal Clusters
Source: Molecules. 2021 Aug 31;26(17):5294. doi: 10.3390/molecules26175294 (PMC8433677; doi:10.3390/molecules26175294)
Supplement: Supplementary file 1 [file molecules-26-05294-s001.zip › molecules-1334710-supplementary.pdf]

molecules

MDPI

Supplementary Materials

Hyper-Cross-Linked Polystyrene as a Stabilizing Medium for Small Metal Clusters

Alexey V. Bykov [1], Galina N. Demidenko [1], Linda Zh. Nikoshvili [1,*] and Lioubov Kiwi-Minsker [2,3,*]

1 Department of Biotechnology, Chemistry and Standardization, Tver State Technical University, A. Nikitina str. 22, 170026 Tver, Russia; bykovav@yandex.ru (A.V.B.); xt345@mail.ru (G.N.D.)
2 Regional Technological Centre, Tver State University, Zhelyabova str. 33, 170100 Tver, Russia
3 Department of Basic Sciences, Ecole Polytechnique Fédérale de Lausanne, ISIC-FBS-EPFL, CH-1015 Lausanne, Switzerland
* Correspondence: nlinda@science.tver.ru (L.Z.N.); lioubov.kiwi-minsker@epfl.ch (L.K.-M.); Tel.: +7-904-005-7791 (L.Z.N.); +41-21-693-3182 (L.K.-M.)

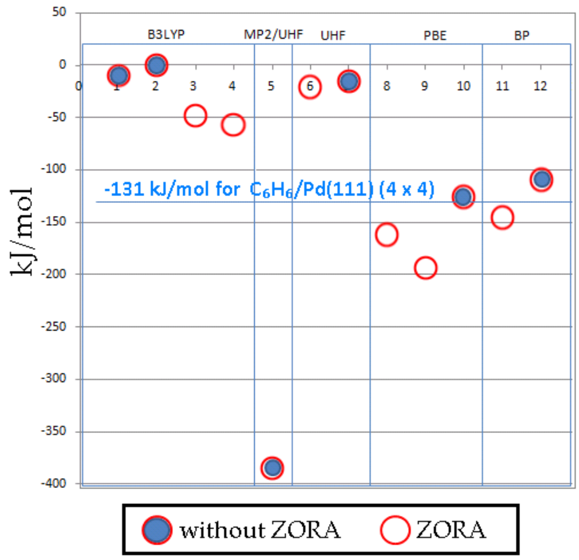

#	1	2	3	4	5	6	7	8	9	10	11	12
Basis set for H and C	ma-def2-TZVP	def2-TZVP	ZORA-def2-TZVP		def2-TZVP	ZORA-def2-TZVP	def2-TZVP	ZORA-def2-TZVP		def2-TZVP	ZORA-def2-TZVP	def2-TZVP
Basis set for Pd	ma-def2-TZVP	def2-TZVP	old-ZORA-TZVP*	ZORA-TZV	def2-TZVP	old-ZORA-TZVP	def2-TZVP	old-ZORA-TZVP		def2-TZVP	old-ZORA-TZVP	def2-TZVP
ECP	Def2-ECP	Def2-ECP	-	-	Def2-ECP	-	Def2-ECP	-		Def2-ECP	-	Def2-ECP

*the designation adopted in the Orca package.

Figure S1. Comparison of different calculation methods and basis sets for Pd_4*C_6H_6 adsorption complex.

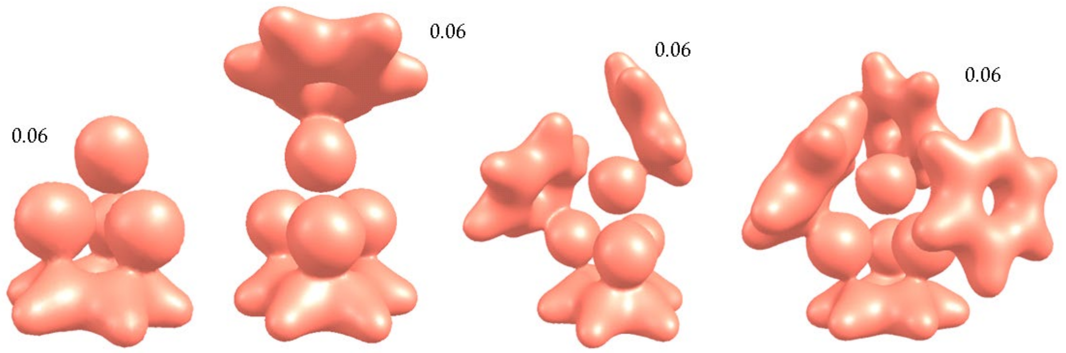

Figure S2. Isosurfaces 0.06 e/Å^3 of the adsorption complexes Pd_4*C_6H_6, Pd_4*2C_6H_6 , Pd_4*3C_6H_6 and Pd_4*4C_6H_6.

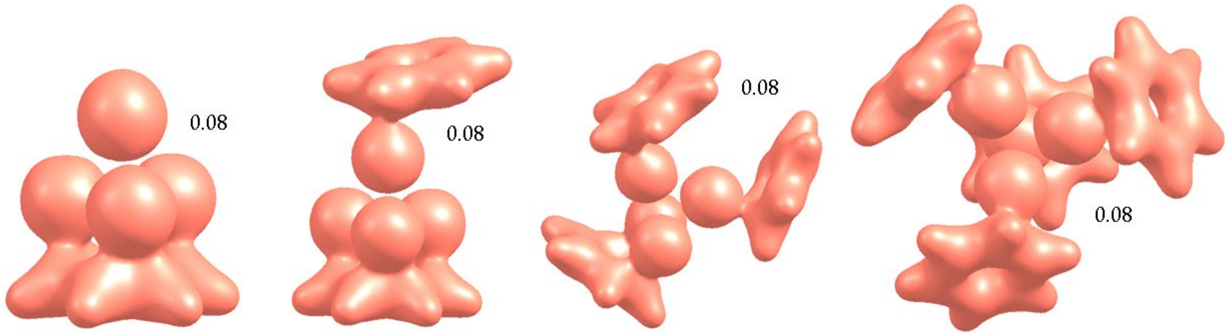

Figure S3. Isosurfaces 0.08 e/Å^3 of the adsorption complexes Pt_4*C_6H_6, Pt_4*2C_6H_6, Pt_4*3C_6H_6 and Pt_4*4C_6H_6.

Table S1. Coordinates of atoms of the optimized structures in the ground state.

**Pd_4 Triplet**			
Pd	−3.088033223	0.266465515	−0.030968796
Pd	−0.932530614	1.626164072	0.012250316
Pd	−0.797942236	−0.980861152	0.025477173
Pd	−1.604830377	0.237694104	2.115442217
**Pd_4*C_6H_6 Triplet**			
Pd	−1.706964666	−0.808877135	−0.908263054
Pd	−0.873301806	1.692200862	−1.065690193
Pd	0.390856980	−0.027116924	0.479089798
Pd	0.560789105	−0.248221038	−2.145864620
C	−2.190701309	−0.911449215	−3.105687265
C	−0.946482732	−0.604225338	−3.784981719
C	−0.497554189	0.742099420	−3.872591004
C	−1.283076491	1.808398336	−3.281779705
C	−2.499961951	1.506866064	−2.611117218
C	−2.959445002	0.133211321	−2.523136497
H	−2.642039354	−1.891200415	−3.271501354
H	−0.511128580	−1.365738279	−4.434408465
H	0.280303283	1.009677928	−4.590037315
H	−1.065594151	2.836248492	−3.577357324
H	−3.212980478	2.304452442	−2.394834906
H	−3.999521660	−0.046486522	−2.245242162
**Pd_4*2C_6H_6 Triplet**			
Pd	−0.128421130	−1.222777711	0.742447449
Pd	1.507416201	0.619124953	1.691480403
Pd	2.274434412	−1.956402545	1.655160924

Pd	2.167781130	−0.670374146	−0.708636787
C	−0.783923037	0.009658876	−0.926266522
C	0.432528072	0.324564380	−1.653852614
C	1.322092541	1.315243624	−1.145740740
C	0.995086924	2.008777370	0.090770407
C	−0.247102762	1.759455153	0.749947735
C	−1.135677600	0.749487078	0.247020043
H	−1.554322878	−0.564057833	−1.447543515
H	0.500215717	0.014516112	−2.699183701
H	2.063065510	1.772379016	−1.805712053
H	1.542784421	2.927686019	0.315123210
H	−0.609594279	2.453988423	1.508844184
H	−2.155099625	0.702457115	0.632514049
C	3.699376819	−3.595818545	2.245055813
C	2.456342275	−3.834151678	2.902450689
C	4.723407650	−2.897947975	2.929632740
H	1.715696108	−4.497919195	2.453636052
H	5.676559866	−2.722036015	2.428986891
C	2.276020608	−3.372805766	4.228201350
C	4.528533705	−2.467205035	4.240948821
H	1.328935864	−3.562251144	4.735327882
H	5.329702758	−1.943890296	4.765043347
C	3.304874148	−2.705117673	4.890134626
H	3.159087925	−2.366941061	5.917140828
H	3.917002998	−4.077035163	1.289644179
**Pd_4*3C_6H_6 Triplet**			
Pd	0.814754029	−1.858542545	2.051587813
Pd	2.000435353	0.725459358	1.723858674
Pd	2.963290096	−1.188985064	3.594544094
Pd	3.162891998	−1.670510837	0.941520052
C	0.447267875	−2.248095257	−0.136773089
C	1.736452372	−2.155011038	−0.764459203
C	2.360782036	−0.879839009	−0.923822924
C	1.655167628	0.325867357	−0.532677907
C	0.383681995	0.238972782	0.061368874
C	−0.208736086	−1.058649428	0.318335565
H	−0.107873445	−3.184354797	−0.207392292
H	2.135379532	−3.027308940	−1.283779671
H	3.206845854	−0.775989967	−1.607240180
H	2.014696226	1.283043159	−0.915866070
H	−0.243182187	1.126697333	0.151322645
H	−1.276482228	−1.092891843	0.549413385
C	4.301183849	−2.885573767	4.246003527
C	3.270865041	−2.909202603	5.226951559
C	5.534819253	−2.260470113	4.563863562
H	2.364518221	−3.488993630	5.046445637
H	6.334219267	−2.249826640	3.821390567
C	3.500563397	−2.343061608	6.497599499
C	5.739205361	−1.698701538	5.822595420
H	2.716152459	−2.392857653	7.255032037
H	6.697706336	−1.233171560	6.059282825

C	4.726646647	−1.747933585	6.796816918
H	4.901442235	−1.323784307	7.786839281
H	4.224771911	−3.511968019	3.356219856
C	1.724944782	2.476967268	3.119449982
C	0.585655669	2.237150762	3.924200500
C	2.945822880	1.788732182	3.420611408
H	−0.349407790	2.750664696	3.693494280
H	3.881114609	2.123916058	2.966335834
C	0.661378906	1.390711169	5.030212477
C	3.018862576	0.944095451	4.584581401
H	−0.221102369	1.236838343	5.653389021
H	4.000703682	0.648825112	4.969717251
C	1.876749846	0.780879294	5.386775005
H	1.946471783	0.193549252	6.302307909
H	1.725300668	3.316185058	2.420922348
**Pd_4*4C_6H_6 Triplet**			
Pd	−2.119250314	−1.002475172	−3.263949617
Pd	−3.150962274	1.705906543	−3.461148240
Pd	−1.117028857	1.007911575	−1.594446332
Pd	−0.614335151	0.984020120	−4.223778816
C	−1.882279674	−1.134099899	−5.690382596
C	−1.075343711	−0.039436770	−6.129562038
C	−1.625474965	1.284174897	−6.152615348
C	−3.001987140	1.496914954	−5.777690454
C	−3.781645974	0.418828373	−5.310258681
C	−3.206899816	−0.906163034	−5.230615347
H	−1.519834807	−2.152682602	−5.828218971
H	−0.138244836	−0.250694179	−6.647656021
H	−1.108486266	2.075549405	−6.698639876
H	−3.479698275	2.439425363	−6.051111157
H	−4.864810008	0.520164025	−5.230338183
H	−3.895675506	−1.748720350	−5.146146305
C	1.080231024	1.016863320	−0.931916194
C	0.402643252	0.134032992	−0.045581303
C	1.394184541	2.327377467	−0.494792424
H	0.288251331	−0.919090713	−0.307769493
H	1.928686637	3.000906956	−1.166669575
C	0.060985515	0.577807298	1.250731625
C	1.048941295	2.745520221	0.788528306
H	−0.437328374	−0.109386316	1.937041418
H	1.308146670	3.752746688	1.120008017
C	0.384895596	1.869661814	1.665956693
H	0.130156529	2.199326321	2.674630768
H	1.523243538	0.632684976	−1.851568200
C	−4.488842045	2.929623320	−2.126852873
C	−5.248660883	2.012254512	−1.363649129
C	−3.124404798	3.168688008	−1.766331118
H	−6.286796018	1.817187053	−1.638129559
H	−2.589520521	4.014124154	−2.202716282
C	−4.698559358	1.403944775	−0.232038510
C	−2.574854216	2.551991745	−0.588054973

H	−5.312767656	0.727655206	0.365610977
H	−1.654769642	2.956663102	−0.152198859
C	−3.389855040	1.699191943	0.179382327
H	−3.001134954	1.284415307	1.109905919
H	−4.989907987	3.579195252	−2.847616423
C	−2.690580277	−3.213294840	−2.774028839
C	−3.348015119	−2.358828771	−1.843736789
C	−1.540030485	−3.930883547	−2.365617988
H	−4.330021354	−1.942093431	−2.072834567
H	−1.043649267	−4.594661198	−3.075511610
C	−2.851991070	−2.270288958	−0.519069785
C	−1.066487851	−3.820947722	−1.064033814
H	−3.370987371	−1.635036235	0.199109344
H	−0.186878681	−4.388155187	−0.755165392
C	−1.733734741	−2.998109078	−0.134124695
H	−1.372313917	−2.937266778	0.893871564
H	−3.175112716	−3.479831856	−3.714438929
**Pd_9 Triplet**			
Pd	−1.425832121	0.203088857	−0.433677126
Pd	0.417942394	2.027536061	0.009661138
Pd	2.128118255	0.216509209	0.976394782
Pd	0.261842088	−1.671769912	0.496053801
Pd	−0.353823537	0.455567937	1.946083270
Pd	1.014003511	−0.122829554	−1.448898699
Pd	1.744321017	−2.709264968	−1.457151921
Pd	3.532407573	−0.829981017	−1.047364863
Pd	2.797506031	−2.367680973	0.933045848
**Pd_9*C_6H_6 Triplet**			
Pd	−1.28591978460323	−0.52501866063557	−1.47995039115077
Pd	−0.10305758561796	1.82675394675646	−1.23915866313424
Pd	2.08544859500528	0.98972299629893	−2.53023003605310
Pd	0.78749984658333	−1.56952517104483	−2.79794858344295
Pd	1.13732378143124	−0.36171259049458	−0.45286755617734
Pd	−0.50995416776784	0.80053717420367	−3.65849497461307
Pd	0.45414049326083	−1.01873561861053	−5.37785360492665
Pd	1.65648655743928	1.33239680840469	−5.13785506359394
Pd	2.99577976237537	−0.92445031472398	−4.15214967193871
C	1.91121219410069	−1.72195111914965	−6.88514939428518
C	3.20772486137465	−1.64088040794610	−6.25158145125925
C	3.85989710105238	−0.37763398643015	−6.12655316370335
C	3.21660152370539	0.81459804695689	−6.62860050648532
C	1.95479264856842	0.72533052424609	−7.29866072201090
C	1.30148589009942	−0.54647097619265	−7.42935852825506
H	1.54924057994772	−2.70608973355471	−7.19059717303345
H	3.78070348028156	−2.56278940828117	−6.13019486100640
H	4.92957189452114	−0.33633015852486	−5.90973035598894
H	3.82088295471811	1.71754091904671	−6.73921173796548
H	1.59394992785636	1.56777559304804	−7.89067108711724
H	0.46306262966779	−0.63998740037269	−8.12127227585848
**Pd_9*Polymer Triplet**			
C	−2.561902376	−2.144673315	1.487610187

H	−1.813552250	−2.293967455	2.291700591
C	−2.041312467	−3.106447502	0.365079194
H	−1.082281397	−2.679952171	0.038681946
H	−1.792762080	−4.055897948	0.862381602
C	−2.416403022	−0.661825073	1.131713889
C	−3.475669044	0.243980348	1.020613758
C	−1.089624167	−0.147123396	1.009738613
C	−3.264177042	1.644456739	0.798470303
H	−4.502036009	−0.085430462	1.178945478
C	−0.854115488	1.222402502	0.683941722
H	−0.255934291	−0.751175498	1.378704082
C	−1.956566714	2.141310666	0.542373980
H	−4.086167630	2.343392180	0.986936455
H	0.139929928	1.643949951	0.866173359
C	−2.894705843	−3.449608678	−0.900371330
H	−3.880955144	−2.962555086	−0.822586515
C	−3.109684650	−4.994694528	−0.940286298
C	−2.253840885	−2.988842422	−2.219235800
H	−2.114931018	−5.463714197	−1.029966740
H	−3.512077712	−5.303974923	0.037104495
C	−3.066400141	−2.680903458	−3.371797843
C	−0.867189848	−3.197336367	−2.464026226
C	−2.469488789	−2.553263255	−4.679058336
H	−4.151710138	−2.798467765	−3.316403314
C	−0.306026723	−3.109800857	−3.742940791
H	−0.218571847	−3.482412771	−1.633954867
C	−1.082037460	−2.777747220	−4.863460977
H	−3.135387306	−2.519139192	−5.546642089
H	0.766900791	−3.272300005	−3.863626004
C	−3.930515086	−2.507362600	2.130370253
H	−4.733406383	−2.225261385	1.427853624
C	−4.148187819	−1.738665745	3.456171371
C	−4.122155350	−3.986432025	2.438527270
H	−3.410715915	−2.065281575	4.204925632
H	−4.045311630	−0.653678065	3.331835335
H	−5.147138539	−1.950131512	3.862983130
C	−5.339711627	−4.612985939	2.134573837
C	−3.151342388	−4.735390771	3.123698087
C	−5.582536044	−5.942363715	2.492046026
H	−6.112691390	−4.044013006	1.611500120
C	−3.382842524	−6.067114389	3.474199751
H	−2.198905861	−4.272809263	3.392984425
C	−4.600780442	−6.677263546	3.160529040
H	−6.541446391	−6.403166570	2.247363622
H	−2.609576815	−6.630685109	3.999549453
H	−4.783568404	−7.716037504	3.439947986
C	−4.019059096	−5.560398086	−2.054237981
H	−3.653528517	−5.175188091	−3.020175735
C	−5.486693760	−5.124078212	−1.893819957
C	−3.903541125	−7.076707096	−2.126834323
H	−5.903471678	−5.488186683	−0.943391710

H	−5.587865451	−4.028698518	−1.902304893
H	−6.106025131	−5.525456835	−2.708584105
C	−3.413783048	−7.696788950	−3.285873098
C	−4.280232010	−7.896347504	−1.049823041
C	−3.304285949	−9.087550858	−3.373725799
H	−3.112150329	−7.076495516	−4.133653806
C	−4.171761951	−9.286151551	−1.131812318
H	−4.658535548	−7.444247040	−0.129969789
C	−3.684485416	−9.888922492	−2.295277281
H	−2.920941921	−9.544792002	−4.288025260
H	−4.469339810	−9.903174706	−0.281622349
H	−3.600533214	−10.975173338	−2.358980985
C	−0.266303828	5.396330011	−5.148536886
H	−1.016197942	6.184943254	−5.337118585
C	−0.370995166	4.493169005	−6.416947852
H	−1.379387312	4.019106975	−6.386973000
H	−0.404055798	5.189046217	−7.269745683
C	−0.687252418	4.787503107	−3.817629996
C	0.099422935	3.839035384	−3.118310908
C	−1.854696147	5.276598861	−3.171056838
C	−0.206188614	3.480681206	−1.760216490
H	1.066418840	3.538096401	−3.524372481
C	−2.180489598	4.890862932	−1.822935750
H	−2.378193726	6.127263818	−3.615575017
C	−1.337403444	4.017400393	−1.096339410
H	0.537233424	2.920322065	−1.193191316
H	−2.965536095	5.432348946	−1.289276885
C	0.698128135	3.400916590	−6.747877194
H	1.598221012	3.594083791	−6.141060664
C	1.094602000	3.564846893	−8.246686530
C	0.285733736	1.955633505	−6.472774849
H	0.184285386	3.426246422	−8.853953767
H	1.410953669	4.608907612	−8.404027749
C	−0.896428859	1.398190475	−7.044585335
C	1.176283015	1.086541898	−5.801170640
C	−1.152665304	−0.027015728	−6.952799183
H	−1.403396718	1.935663038	−7.856191718
C	0.932699557	−0.321509391	−5.673257519
H	2.163691669	1.470310231	−5.534971297
C	−0.264303086	−0.897781580	−6.248874043
H	−1.907884346	−0.452887252	−7.617403773
H	1.784173110	−0.992076827	−5.497148228
C	1.106938445	6.137977796	−5.029123783
H	1.893228167	5.376229448	−4.888365930
C	1.147004443	7.078699228	−3.807820474
C	1.467397902	6.928827942	−6.280035162
H	0.353338410	7.838316615	−3.874179960
H	1.007615036	6.525474227	−2.869220266
H	2.110648941	7.605118874	−3.761077539
C	2.719509839	6.763294804	−6.889613296
C	0.598038073	7.893872565	−6.814904538

C	3.094812953	7.530427104	−7.996683246
H	3.414263470	6.023027684	−6.485171343
C	0.963946616	8.658154094	−7.924564820
H	−0.380087318	8.057823412	−6.356745476
C	2.215654154	8.480079261	−8.521015948
H	4.078619708	7.386573672	−8.447461581
H	0.269264372	9.399446927	−8.323818948
H	2.503468122	9.080134611	−9.385630079
C	2.189582982	2.619130507	−8.787608458
H	1.896650191	1.587390330	−8.529717102
C	3.567815044	2.891452744	−8.159827512
C	2.252384228	2.690457180	−10.306681409
H	3.912686212	3.910188742	−8.390704222
H	3.540817531	2.785792301	−7.065738208
H	4.319374378	2.188203121	−8.545745579
C	2.610205768	3.874655101	−10.972666128
C	1.942226740	1.565804664	−11.085284363
C	2.655026352	3.930844170	−12.367180103
H	2.853650451	4.769836585	−10.396148457
C	1.987067654	1.615644043	−12.481544254
H	1.660159188	0.635483110	−10.585639717
C	2.343993452	2.800451017	−13.128589466
H	2.933630299	4.862990194	−12.862606093
H	1.741616963	0.725812909	−13.064447506
H	2.379868180	2.843981739	−14.218403415
C	−0.455301112	−2.417779770	−6.205867259
H	0.511023322	−2.930582893	−6.332968147
H	−1.111277788	−2.734354351	−7.031787077
C	−1.672909171	3.626428194	0.337690248
H	−2.549575846	4.207438955	0.660699046
H	−0.832450247	3.916217461	0.995232969
Pd	−3.326190510	1.571149112	−1.373849454
Pd	−3.347660947	3.565960698	−3.277376884
Pd	−1.262887058	1.981920408	−3.141101246
Pd	−2.745234417	−0.763098880	−2.442910351
Pd	−0.563217052	0.160021787	−1.198067226
Pd	−4.114094872	1.088570678	−3.867986081
Pd	−1.979512322	−0.268415394	−4.915541661
Pd	−2.359734704	2.272644518	−5.555341222
Pd	0.568458788	0.213902153	−3.634962382
**Pt_4 Triplet**			
Pt	−1.653639782	−0.790020607	−0.961968067
Pt	−0.842737317	1.638640360	−1.118754516
Pt	0.332316746	−0.011613008	0.472336039
Pt	0.537420941	−0.244298552	−2.200145051
**Pt_4*C_6H_6 Triplet**			
Pt	−1.724979520	−0.800879091	−0.940396846
Pt	−0.892818855	1.692575122	−1.098150179
Pt	0.372961523	−0.023768369	0.444784209
Pt	0.537210865	−0.242444821	−2.172841965
C	−2.170723508	−0.942118855	−3.046648023

C	−0.897178742	−0.628062876	−3.741327411
C	−0.442298539	0.736719307	−3.830962111
C	−1.245763021	1.827643718	−3.225855394
C	−2.482144060	1.522198473	−2.550489400
C	−2.952000097	0.117904836	−2.460955918
H	−2.655980186	−1.885884372	−3.304648656
H	−0.530555288	−1.361196581	−4.463034716
H	0.260816996	1.013704989	−4.619410873
H	−1.082162504	2.837165029	−3.609003006
H	−3.233431418	2.306054919	−2.432500333
H	−4.017756646	−0.039771428	−2.281962377
**Pt_4*2C_6H_6 Triplet**			
Pt	−0.273235560	−1.203563609	0.789740941
Pt	1.382805257	0.532176619	1.753476665
Pt	2.111841321	−2.037650409	1.640975125
Pt	2.134525102	−0.752532283	−0.659474351
C	−0.757867935	−0.020987180	−0.904131633
C	0.512822535	0.246778320	−1.612968280
C	1.437496610	1.219290091	−1.076835526
C	1.096692592	1.926398956	0.178229081
C	−0.188335227	1.731183544	0.812245115
C	−1.129114055	0.745450082	0.264338884
H	−1.549363420	−0.496703347	−1.490718710
H	0.533732085	0.019063171	−2.682454461
H	2.138698804	1.722486340	−1.748128157
H	1.640781830	2.854545122	0.376266866
H	−0.582069876	2.500418147	1.478592583
H	−2.180426856	0.828447598	0.544010997
C	3.670740970	−3.543210403	2.195398678
C	2.457208367	−3.859345652	2.894746682
C	4.672875296	−2.782992059	2.856312803
H	1.770705231	−4.606017733	2.491470119
H	5.598029510	−2.550197122	2.327597440
C	2.296111157	−3.408084624	4.233188293
C	4.488342579	−2.360486818	4.169730960
H	1.381134193	−3.658096212	4.771602425
H	5.269379958	−1.786147882	4.669617105
C	3.299912554	−2.673685126	4.857433848
H	3.166240221	−2.341714928	5.887915441
H	3.907141097	−4.044216269	1.254396756
**Pt_4*3C_6H_6 Triplet**			
Pt	0.780473903	−1.763571766	2.070704256
Pt	1.940830469	0.628323570	2.584515976
Pt	3.026457800	−1.541148993	3.477541273
Pt	2.947921584	−1.160128961	0.844971333
C	0.556945599	−2.628985348	0.123452035
C	1.800987677	−2.281541110	−0.585282536
C	1.961228083	−0.951632614	−1.119856207
C	0.859863833	−0.015884880	−1.026637420
C	−0.297221626	−0.337866020	−0.368523537
C	−0.473390546	−1.628172637	0.265894958

H	0.306247299	−3.688331363	0.218437336
H	2.411288748	−3.101365504	−0.972210699
H	2.719511187	−0.774870092	−1.886437694
H	0.966092760	0.961239130	−1.500113784
H	−1.115327529	0.382149669	−0.318147158
H	−1.491081460	−1.944981998	0.506783480
C	4.529589867	−3.099609394	3.908061199
C	3.313143143	−3.418987028	4.602766268
C	5.542981466	−2.367908685	4.595011687
H	2.619715261	−4.151805528	4.189759404
H	6.470720514	−2.132541237	4.071608374
C	3.158474646	−2.987705674	5.952867282
C	5.372469762	−1.988570357	5.922152395
H	2.237371606	−3.233445524	6.483207271
H	6.165843507	−1.444639514	6.436700681
C	4.177646916	−2.298183666	6.602245080
H	4.048943015	−1.993243373	7.641629743
H	4.768649381	−3.588940563	2.963693802
C	2.503465033	2.652540532	3.203773691
C	1.193303578	2.342902025	3.723872329
C	3.615561061	2.667317346	4.101658557
H	0.300281843	2.610667883	3.150182936
H	4.603663096	2.923711194	3.716115278
C	1.051496208	2.054700548	5.115420245
C	3.439312180	2.379734065	5.441219580
H	0.058666336	1.838114813	5.513018739
H	4.295487435	2.398141071	6.117427498
C	2.152375418	2.071371522	5.950488300
H	2.031185428	1.854435458	7.012972349
H	2.602785785	3.161543886	2.240033571
**Pt_4*4C_6H_6 Triplet**			
Pt	−2.625541361	−1.105013766	−2.193404523
Pt	−3.169820711	1.361853839	−1.656796619
Pt	−0.671474112	0.605102807	−1.433730484
Pt	−1.722075728	0.839104528	−3.773428695
C	−0.477965599	−0.788208558	−5.993819008
C	−0.307052247	0.537584394	−5.500253006
C	−1.314626742	1.525581127	−5.780018742
C	−2.496938074	1.114630564	−6.466471280
C	−2.631124838	−0.189545901	−6.934537450
C	−1.610891061	−1.137850981	−6.714246062
H	0.304351303	−1.525467619	−5.807146868
H	0.675908750	0.849620024	−5.146440695
H	−1.092059171	2.590537643	−5.675937072
H	−3.277723538	1.850865368	−6.662594825
H	−3.531779473	−0.476666527	−7.479366418
H	−1.720281829	−2.152481111	−7.099459557
C	1.524663451	1.030529702	−1.160245000
C	1.089831648	−0.082520985	−0.363026564
C	1.604807903	2.324333218	−0.571250183
H	1.268786127	−1.109117316	−0.685759158

H	1.967113572	3.157656448	−1.175492920
C	0.665961161	0.174741609	0.976379879
C	1.254391688	2.525295295	0.757992885
H	0.304141832	−0.656997253	1.581920849
H	1.338309546	3.518778570	1.200210843
C	0.767009661	1.450362215	1.527121086
H	0.465812745	1.615194635	2.562790288
H	2.001295332	0.855018528	−2.125127385
C	−5.251589239	1.949011698	−1.020341982
C	−5.154219059	2.079765789	0.392539895
C	−4.666935354	2.956398887	−1.859199459
H	−5.613612318	1.319839619	1.026589355
H	−4.920271518	3.014352513	−2.918337249
C	−4.513813937	3.174260329	0.966806062
C	−3.965660627	4.032085987	−1.239460779
H	−4.468039506	3.269225641	2.052380730
H	−3.502246522	4.791563949	−1.870434039
C	−3.911667927	4.147479560	0.148376822
H	−3.392858718	4.994234977	0.599801893
H	−5.928589717	1.208559642	−1.447214599
C	−3.881299406	−2.830999500	−2.352132333
C	−3.686645047	−2.607564605	−0.939272538
C	−2.990324337	−3.736817555	−3.011622132
H	−4.442147571	−2.064059280	−0.370821976
H	−3.094400225	−3.888920735	−4.086858913
C	−2.731435574	−3.396228968	−0.226818579
C	−2.039641604	−4.454106997	−2.294467932
H	−2.643691249	−3.268043443	0.853379256
H	−1.380768667	−5.148564472	−2.818595174
C	−1.932349653	−4.309781884	−0.890703733
H	−1.205831957	−4.907545035	−0.338389259
H	−4.803786484	−2.524455562	−2.849294999
**Pt_9 Triplet**			
Pt	−1.374765299	0.133862793	−0.467924480
Pt	0.470215488	2.009826562	−0.047020522
Pt	2.166632883	0.284705885	0.975656009
Pt	0.154335117	−1.761930620	0.513337440
Pt	−0.247258291	0.379751727	1.932289416
Pt	1.064854740	−0.166199147	−1.366783254
Pt	1.598448886	−2.709771577	−1.448683990
Pt	3.586421838	−0.688036521	−0.989670079
Pt	2.697599849	−2.281033462	0.872945691
**Pt_4*Polymer Triplet**			
C	−1.884084000	−2.455890000	0.967475000
H	−1.067209000	−2.425436000	1.709579000
C	−1.603966000	−3.734515000	0.138646000
H	−0.536298000	−3.731995000	−0.127420000
H	−1.746173000	−4.589120000	0.817686000
C	−1.793369000	−1.105024000	0.232544000
C	−2.894307000	−0.553652000	−0.521527000
C	−0.823937000	−0.132836000	0.740379000

C	−3.093730000	0.858330000	−0.644832000
H	−3.754362000	−1.191859000	−0.735129000
C	−1.019553000	1.280740000	0.536109000
H	−0.144063000	−0.464686000	1.528550000
C	−2.192958000	1.779553000	−0.138279000
H	−3.984199000	1.208545000	−1.171510000
H	−0.450663000	1.979957000	1.158349000
C	−2.425339000	−3.982192000	−1.155099000
H	−3.404298000	−3.481303000	−1.067010000
C	−2.686134000	−5.502846000	−1.316403000
C	−1.726483000	−3.399505000	−2.383719000
H	−1.713413000	−6.020235000	−1.360177000
H	−3.180430000	−5.864993000	−0.398586000
C	−2.425202000	−2.593582000	−3.356611000
C	−0.465948000	−3.943398000	−2.807774000
C	−1.870955000	−2.380449000	−4.654766000
H	−3.479496000	−2.355391000	−3.194868000
C	0.040734000	−3.725809000	−4.078822000
H	0.093882000	−4.574912000	−2.114989000
C	−0.642748000	−2.920117000	−5.023274000
H	−2.423672000	−1.752377000	−5.356650000
H	1.005525000	−4.161108000	−4.349360000
C	−3.207580000	−2.557352000	1.802417000
H	−4.053460000	−2.605622000	1.094960000
C	−3.420844000	−1.326439000	2.705411000
C	−3.267058000	−3.823733000	2.645816000
H	−2.582693000	−1.206391000	3.408563000
H	−3.506222000	−0.401416000	2.120078000
H	−4.338719000	−1.442376000	3.298645000
C	−4.322457000	−4.735060000	2.494519000
C	−2.299244000	−4.095531000	3.627547000
C	−4.411119000	−5.881355000	3.290045000
H	−5.090582000	−4.539360000	1.741668000
C	−2.379617000	−5.240708000	4.421597000
H	−1.466874000	−3.403950000	3.776271000
C	−3.437305000	−6.139926000	4.256560000
H	−5.244233000	−6.573280000	3.152786000
H	−1.613798000	−5.431580000	5.175754000
H	−3.501634000	−7.034411000	4.878101000
C	−3.525695000	−5.929370000	−2.542527000
H	−3.053057000	−5.495445000	−3.439728000
C	−4.973416000	−5.412865000	−2.474230000
C	−3.480372000	−7.441025000	−2.713145000
H	−5.482142000	−5.770364000	−1.566331000
H	−5.012309000	−4.314283000	−2.466569000
H	−5.553223000	−5.759621000	−3.341236000
C	−2.796972000	−8.013843000	−3.795864000
C	−4.100228000	−8.303881000	−1.794243000
C	−2.733475000	−9.400357000	−3.960821000
H	−2.306171000	−7.359859000	−4.521065000
C	−4.039275000	−9.689932000	−1.953355000

H	−4.639993000	−7.890447000	−0.939214000
C	−3.355750000	−10.244871000	−3.039026000
H	−2.197690000	−9.820959000	−4.813980000
H	−4.529615000	−10.340464000	−1.226508000
H	−3.309154000	−11.327873000	−3.164602000
C	0.105078000	5.310524000	−5.325402000
H	−0.728533000	5.867117000	−5.790374000
C	0.377845000	4.146520000	−6.334268000
H	−0.556012000	3.567502000	−6.365794000
H	0.489076000	4.599400000	−7.332626000
C	−0.470521000	4.807288000	−4.003220000
C	0.350683000	4.469097000	−2.866179000
C	−1.853342000	4.702644000	−3.864626000
C	−0.282133000	3.938391000	−1.675325000
H	1.369250000	4.857206000	−2.797284000
C	−2.469574000	4.237151000	−2.678912000
H	−2.488651000	4.994388000	−4.704925000
C	−1.719188000	3.833275000	−1.588818000
H	0.287050000	3.928415000	−0.741628000
H	−3.559852000	4.184029000	−2.633674000
C	1.571788000	3.172559000	−6.093499000
H	1.951301000	3.331736000	−5.067421000
C	2.713829000	3.472427000	−7.108488000
C	1.171861000	1.701106000	−6.174833000
H	2.337418000	3.227679000	−8.116017000
H	2.905558000	4.557287000	−7.109407000
C	0.219986000	1.233372000	−7.081362000
C	1.827907000	0.735872000	−5.356784000
C	−0.146150000	−0.124280000	−7.135379000
H	−0.275768000	1.931327000	−7.758338000
C	1.514776000	−0.661489000	−5.448768000
H	2.701075000	1.041657000	−4.779611000
C	0.460993000	−1.082728000	−6.325800000
H	−0.927930000	−0.436662000	−7.832217000
H	2.227879000	−1.400708000	−5.069420000
C	1.252078000	6.341738000	−5.145339000
H	2.119913000	5.829625000	−4.695379000
C	0.824069000	7.484770000	−4.196570000
C	1.740577000	6.975467000	−6.443327000
H	−0.038341000	8.023476000	−4.617938000
H	0.537844000	7.110730000	−3.204669000
H	1.640946000	8.209184000	−4.071619000
C	3.105048000	7.267987000	−6.599566000
C	0.864982000	7.381545000	−7.462520000
C	3.582265000	7.934314000	−7.730622000
H	3.805027000	6.972801000	−5.813188000
C	1.336757000	8.041922000	−8.600258000
H	−0.205141000	7.183128000	−7.373070000
C	2.697901000	8.321835000	−8.740265000
H	4.648151000	8.151400000	−7.822698000
H	0.634560000	8.340473000	−9.380921000

H	3.066133000	8.838345000	−9.627996000
C	4.049016000	2.728574000	−6.886253000
H	3.825295000	1.653683000	−6.785271000
C	4.764020000	3.184519000	−5.600593000
C	4.961795000	2.861142000	−8.096144000
H	4.999934000	4.258762000	−5.643625000
H	4.142843000	3.012158000	−4.709181000
H	5.709165000	2.640705000	−5.461932000
C	5.360797000	4.116334000	−8.584610000
C	5.438519000	1.717552000	−8.753947000
C	6.207738000	4.222169000	−9.689862000
H	4.997808000	5.026778000	−8.102672000
C	6.287337000	1.817362000	−9.859894000
H	5.134581000	0.731648000	−8.392891000
C	6.676814000	3.072512000	−10.331996000
H	6.502008000	5.208585000	−10.053709000
H	6.643669000	0.911293000	−10.353831000
H	7.337916000	3.155662000	−11.196300000
C	0.013312000	−2.533902000	−6.338694000
H	0.878007000	−3.194126000	−6.516595000
H	−0.684225000	−2.682656000	−7.177940000
C	−2.375012000	3.278587000	−0.331782000
H	−3.450948000	3.506602000	−0.372410000
H	−1.965700000	3.802393000	0.549611000
Pt	−1.266238000	−1.269137000	−1.983143000
Pt	0.389744000	2.343357000	−2.931079000
Pt	0.266349000	0.567395000	−1.059688000
Pt	0.449172000	−0.124400000	−3.628494000
